# Vaginal colonization, vertical transmission rate, antimicrobial susceptibility profile, and associated factors of potential neonatal pathogens among pregnant women at public health facilities of Northeast Ethiopia

**DOI:** 10.3389/fpubh.2025.1475357

**Published:** 2025-02-05

**Authors:** Getnet Shimeles, Alemu Gedefie, Hilina Motbainor, Chalachew Genet

**Affiliations:** ^1^Gishe Rabel Health Center, Debre Birhan, Ethiopia; ^2^Department of Medical Laboratory Sciences, College of Medicine and Health Sciences, Bahir Dar University, Bahir Dar, Ethiopia; ^3^Department of Medical Laboratory Sciences, College of Medicine and Health Sciences, Wollo University, Dessie, Ethiopia; ^4^Institute of Biotechnology, Bahir Dar University, Bahir Dar, Ethiopia

**Keywords:** vaginal colonization, vertical transmission rate, antimicrobial susceptibility antibiotic resistance, multi-drug resistance, neonatal disease, pregnant women, vertical transmission

## Abstract

**Background:**

Vaginal colonization by pathogenic bacteria increases the risk of bacterial infections such as sepsis, which is associated with high neonatal mortality. More than half a million newborn deaths occur globally each year due to infections that lead to sepsis. However, the problem is worsening in Ethiopia the evidence of vaginal colonization and vertical transmission is scarce.

**Methods:**

A healthcare facility-based cross-sectional study was conducted in Dessie town from April 1 to June 30, 2023, among 348 pregnant women and their newborns. Socio-demographic, clinical, and related data were collected using a pre-tested semi-structured questionnaire. Vaginal swab samples from pregnant women and pooled external ear, nasal area, and umbilical swab samples from the newborns were collected and transported using Amies transport media. Samples were inoculated into blood agar, Todd Hewitt selective enrichment broth, and MacConkey agar for bacterial isolation, and Sabouraud Dextrose Agar and CHROM Agar for Candida species isolation. The antimicrobial susceptibility was performed on isolates using the Kirby-Bauer disc diffusion technique. Data was analyzed by SPSS version 25.0. Logistic regression model was used to identify the associated factors. Finally, variables with *p* < 0.05 and their 95% confidence interval were considered statistically significant.

**Results:**

A total of 348 pregnant women attending vaginal delivery were included in the study. The maternal colonization and vertical transmission rates were 55.5% (193/348) and 53.9% (104/348), respectively. The most frequent potential pathogen among pregnant women was *Escherichia coli* (27.6%), followed by *Candida* spp. (14.1%), and *Klebsiella* spp. (6%). Similarly, the predominant isolates in the newborns were *E. coli* (16.4%), *Candida* spp. (6.3%), and *Klebsiella* spp. (3.6%). The overall multidrug resistance levels of potential pathogens were 37.3%. Living with domestic animals (*p* = 0.001), having premature rupture of membrane (*p* = 0.010) and history of urinary tract infection (*p* = 0.013) were significantly associated with maternal colonization. Potential pathogen colonization newborn was significantly associated with duration of labor (*p* = 0.024) and low birth weight (*p* < 0.001).

**Conclusion:**

The finding of the present study revealed that vaginal colonization and vertical transmissions of potential pathogens and their antimicrobial resistance is still a significant problem. This alarms the urgency of evidence based-intervention to improve maternal and neonatal health.

## 1 Introduction

Colonization of the vaginal compartment by pathogenic bacteria can lead to high perinatal mortality (1). Bacterial vaginitis doubles the chance of preterm birth in women experiencing labor before 37 weeks of pregnancy, and it raises the chance of postpartum maternal infection and late miscarriage (2). Thus, vaginal colonization may lead to neonatal infections like sepsis and other adverse outcomes. Moreover, the first month of child life is the most risky for infection and related complications including neonatal deaths (3). Preterm birth, intrapartum-related complications, infections, and birth defects are the major causes of most neonatal deaths (4).

Globally, almost 2.4 million newborns died in 2020, which is increased from 40% in 1990 in to 47% (4). According to the Global Sepsis Alliance estimates, infections resulting in sepsis account for around 25% of all neonatal deaths in South Asia and Sub-Saharan Africa (SSA), accounting for over one-fifth of the 2.7 million infant deaths that occur worldwide annually (5). Ethiopia has the greatest rate of recoded newborn deaths among Sub-Saharan African countries, making the issue there considerably worse. The 2019 Ethiopian Demographic and Health Survey shows that the government is not doing well in achieving the Sustainable Development Goals target of 12 deaths per 1,000 live births or fewer by 2030, as evidenced by the 30 neonatal deaths per 1,000 live births, and an incidence of 2,824 neonatal sepsis cases per 100.000 live births with 17.6% sepsis related mortality (6, 7). Furthermore, the pooled prevalence of neonatal sepsis in Ethiopia was 45%, of which 64% was found in the Amhara region (8).

Vaginal colonization by pathogenic microorganisms, particularly multidrug-resistant organisms (MDROs), poses a serious risk for vertical transmission to newborns either before or during delivery cause about 80% of cases of early-onset neonatal sepsis (EOS) to arise in the first week of life (9). Depending on the season and geographical location, several microorganisms may be implicated in EOS. For example, in low and middle-income countries (LMICs), bacterial neonatal sepsis is most commonly caused by Gram-negative bacteria, including *Acinetobacter baumannii*, *E. coli*, and *Klebsiella pneumoniae* (10), while in high-income countries, GBS and *E. coli* are the most common (11). However, it wasn’t clarified whether vaginal colonization was the actual etiology of EOS; *Acinetobacter* spp., *Citrobacter* spp., *E. coli*, *S. aureus*, *Enterococcus* spp., *Proteus mirabilis*, and *Klebsiella* spp. were among the bacteria that were identified as the etiological agents of EOS in previous research carried out in Ethiopia (12–15). Generally, GBS and *Escherichia coli* (*E. coli*) are most frequently involved in neonatal sepsis, which account for the majority of infections shared (16). Depending on characteristics including gestational age, socioeconomic background, and the types of bacteria being investigated, different bacteria have different levels of colonization (17, 18). The emergence of MDROs has complicated the treatment of neonatal sepsis, resulting in increased mortality rates globally.

Bacterial organisms that cause neonatal sepsis have become more resistant to commonly used antibiotics. Antibiotic resistance is rapidly spreading and has become a significant problem in the treatment of infectious diseases. Antimicrobial resistant (AMR) bacteria are considered to be clinically most important for human health and are earmarked for surveillance. When antibiotic resistant bacteria colonize the female genital tract, they may be transmitted to the neonate, causing local or systemic neonatal infections that can be difficult to treat with conventionally available antimicrobials (19). Multidrug-resistant organisms spread is associated with increased costs as well as higher morbidity and mortality rates. Hence, one of the major worldwide health concerns is the spread of diseases caused by bacterial pathogens resistant to antibiotics (20). Pathogens frequently resistant to widely prescribed antibiotics are the cause of most invasive infections in neonates (10). These infections are linked to longer hospital stays, a higher chance of complications, and a higher risk of death (21). Reducing the burden of infection with pathogenic antimicrobial resistant bacteria has been prioritized in the World Health Organization’s recently released Global Priority Pathogens List. Fighting the spread of AMR is also listed as one of the top priorities in the UN General Assembly’s 2030 agenda for sustainable global health development (22). Therefore, considering the availability of limited new drug options, antimicrobial stewardship, especially concerning antibiotics in pregnancy, must be optimized.

However, the problem exists and continues in a catastrophic manner in sub-Saharan Africa, particularly in Ethiopia. There is a shortage of up-to-date data on the vaginal colonization rates during gestation, the vertical transmission rate to neonates, the antimicrobial susceptibility profiles impacting treatment, and relevant associated factors for colonization and/or vertical transmission. Furthermore, there is scarcity of evidences in vaginal colonization and vertical transmission in Ethiopia. Thus, this study can show the correlation between maternal colonization and vertical transmission. So as to significantly contribute to the prevention of neonatal sepsis and the improvement of maternal and neonatal health. Furthermore, pregnant women do not have a standard thorough screening protocol for vaginal colonization, and most research studies focus on a single bacterial vaginal colonization and vertical transmission that results in neonatal sepsis. Studying the vital role of vaginal colonization with potential neonatal infections, its transmission from the mother to the infant, and its association with different factors is crucial, especially in light of the lack of available evidence. Therefore, the aim of the present study was to determine the magnitude of vaginal colonization, vertical transmission rate, antimicrobial susceptibility profile of bacterial isolates, and associated factors of potential neonatal pathogens among pregnant women at public health facilities in northeastern Ethiopia.

## 2 Methods and materials

### 2.1 Study area, design and period

This facility-based cross-sectional study was conducted in Dessie Town public health facilities from April 1 to June 30, 2023. Dessie town is 401 kilometers away from Addis Ababa, Ethiopia’s capital. The catchment total population is estimated to be 257,126, of which females are 49.5% and reproductive-age women are estimated to be 23.47%. Dessie town has seven public health centers and one specialized hospital, i.e., Dessie Comprehensive Specialized Hospital (DCSH). The DCSH obstetrics and gynecology ward has 707 healthcare workers, including 16 gynecologists, 3 general physicians, 37 MSc midwives, 13 BSc midwives, and 10 cleaners, whereas the Dessie Health Center (DHC) obstetrics and gynecology ward has 5 healthcare workers, including 1 MSc midwife, 2 BSc midwives, and 2 diploma midwives. In 2022, a total of 13,177 (661 from DHC and 12,446 from DCSH) delivery services were documented. Thus, for this study, DCSH and DHC were selected based on their high client flow for delivery services.

### 2.2 Population and eligibility

Among pregnant women who were attending their antenatal care (ANC) service in the selected health facilities, those pregnant women who were admitted at DCSH and DHC for vaginal delivery service and their newborns during the study period were included in the study. On the contrary, pregnant women who were taking antibiotics within 2 weeks prior to recruitment to the study and those who gave birth via cesarean-section delivery were excluded.

### 2.3 Sample size determination and sampling technique

The sample size was determined using a single population proportion formula by taking the 34.7% prevalence of potential neonatal disease-causing bacteria in pregnant women done in Hawassa (23), a 0.05 margin of error (d), and a 95% confidence interval.


$$n=\frac{\left(Za/2\right)2\left(P\left(1-P\right)\right)}{d2}$$

Where n is the minimum sample size required for the study, Z_a/2_ is 1.96 (95% confidence interval), P is the proportion of the problem (0.347), and d is the margin of error (5%).


$$n=\frac{\left(1.96\right)2\left(0.347\left(1-0.347\right)\right)}{\left(0.05\right)2}=\frac{0.8705}{0.0025}=348$$

The required sample size was 348 pregnant women and their newborn pairs. The sample size was proportionally allocated into DCSH and DHC based on their last year March, April and May, 2022 delivery service performance. Thus, 255 and 93 pregnant women and their newborn pairs were taken from DCSH and DHC, respectively. A convenience sampling technique was used to select the study participants, where samples were collected sequentially from pregnant mothers and their newborns who met the inclusion criteria until the sample size was reached.

### 2.4 Study variables

The outcome variables of this study were vaginal colonization and vertical transmission of potential neonatal pathogens, categorized as 1 = yes and 0 = no. Moreover, socio-demographic characteristics (age, residence, marital status, educational status, occupation, source of drinking water, domestic animal in the house, sex newborn, weight of newborn, 5th minute APGAR score, and status of newborn at delivery), obstetrics-related characteristics (gestational age, number of current ANC visit, history of antibiotic use during the current pregnancy, current gestational hyperbaton, current gestational DM, type of gravidia, history of abortion, history of still birth, premature rupture of membrane, duration of labor, fever during labor, and meconium stained amniotic fluid) and clinical characteristics (history of hospitalization in the past 3 months, HIV status, syphilis status, history of urinary tract infection at current pregnancy, presence of vaginal discharge, and history of sexually transmitted infection at current pregnancy) were an explanatory variables.

### 2.5 Data collection techniques and procedure

#### 2.5.1 Demographic and clinical data collection

A pre-tested semi-structured questionnaire was used to collect demographic, obstetric, and clinically related data from pregnant women and their newborns using a face-to-face interview by attending midwives, and it was complemented with a medical record review after obtaining informed consent and assent.

#### 2.5.2 Specimen collection and transportation

Swabs were taken from the lower vagina region of pregnant mothers during labor by the attending midwife using a sterile cotton swab in accordance with the recommendations of the Centers for Disease Control and Prevention (CDC) (24) and the American College of Obstetricians and Gynecologists (25). Despite, it differs from the CDC’s recommendation, a speculum was utilized during the specimen collection process to ensure optimal visibility and accuracy, and minimize contamination that enhance the sample quality. A sterile speculum without lubricant was inserted in to the vagina followed by insertion of swabs about halfway between the introitus and cervix. Additionally, the labia were carefully separated to avoid cross contamination due to contact from the perineal region. Then, the swab was gently pressed toward the vaginal walls and rotated to ensure that it was thoroughly coated. The midwives took caution when removing the swab to avoid contact with the skin and the anal area. Similarly, from newborns, pooled swabs from the external ear, nasal area, and umbilical area were collected in parallel from the newborn within 15 min after delivery by attending midwife in order to assure vertical transmission. Then, both swabs were placed in amies transport medium without charcoal (OXOID, UK) and transported to the microbiology laboratory of the Amhara Public Health Institute (APHI) Dessie branch for analysis.

#### 2.5.3 Specimen processing and identification

A swab was inoculated on MacConkey and blood agar (Oxoid, Basingstoke, UK) and then incubated aerobically at 37°C for 24 h. From those plates, one representative sample of each morphologically distinct colony was picked and streaked onto new agar plates, and colonies from this sub-culture were used for further species identification and characterization (26). Bacterial isolates were characterized using hemolysis pattern, colony morphology, Gram stain, and a panel of biochemical tests (glucose and lactose fermentation, hydrogen sulfide production, indole production, motility tests, urease production, citrate utilization, and triple sugar iron agar tests for Gram negatives and catalase and coagulase for Gram positives) (27). Moreover, the swabs were placed in Todd Hewitt selective enrichment broth (THB) (Oxoid, UK) supplemented with gentamicin (8 μg/mL) and nalidixic acid (15 μg/mL) to identify GBS following incubation for 24 h at 37°C. When growth (turbidity) was observed, it was sub-cultured on 5% Defibrinated Sheep-Blood Agar (Oxoid, UK) and incubated for 24 h at 37°C in a 5% CO_2_ atmosphere. Flat colonies with narrow beta-hemolysis were considered presumptive GBS and subjected to Gram stain and catalase tests. As a final identification of GBS, all gram-positive cocci and catalase negative isolates were tested for the CAMP factor. Furthermore, *Candida* species was identified by inoculating the swab on Sabouraud Dextrose Agar (SDA) (Oxoid, UK), and incubated at 37°C for 24–48 h. After growth was observed on SDA, white, creamy colonies were identified and streaked on a CHROM agar *Candida* plate (BD Company, Belgium), which was incubated at 37°C for 48 h. Finally, different *Candida* species were identified based on the reaction between specific enzymes of different *Candida* species and a chromogenic substrate, which results in the formation of different colony colors. Green colonies were identified as *C. albicans* and pink colonies with a whitish border were identified as *C. krusei* (28).

#### 2.5.4 Antimicrobial susceptibility testing

Antimicrobial susceptibility testing was done by using the modified Kirby Bauer disc diffusion method based on the Clinical and Laboratory Standards Institute guidelines (CLSI) (29). About 3–5 pure colonies from nutrient agar were taken and homogenously emulsified in 5 mL of tryptone-soya broth. The suspension was incubated at 37°C until the turbidity of the suspension matched with a 0.5 McFarland standard and it was inoculated over the entire surface of the Mueller Hinton agar plate using a sterile swab. Then, the selected antimicrobial disks were put on the inoculated plates and incubated at 37°C for 16–18 h. Antimicrobial agents were selected based on CLSI recommendations and local (Ethiopian) prescription practice. The antimicrobials (Oxoid Ltd) that were used for bacterial susceptibility testing were ampicillin (AMP: 10 μg), erythromycin (E: 15 μg), chloramphenicol (30 μg), Trimethoprim-sulfamethoxazole (SXT: 1.25/23.75 μg), cefuroxime (CRX: 30 μg), ceftriaxone (CTR: 30 μg), cefoxitin (FOX: 30 μg), Amoxicillin-clavulanate (AMC: 20/10 μg), penicillin (P: 10 units), gentamicin (GEN: 10 μg), meropenem (MEM: 10 μg), imipenem (IMP: 10 μg), amikacin (AK: 30 μg), ceftazidime (CAZ: 30 μg), Aztreonam (ATM: 30 μg), cefazolin (CZ: 30 μg), ciprofloxacin (CIP: 5 μg), tetracycline (TE: 30 μg), and clindamycin (DA: 2 μg). Then, the diameters of zones of inhibition were measured using a digital caliper and the identified isolates were reported as sensitive, intermediate, and resistant according to the 2022 CLSI guideline.

### 2.6 Operational definitions

- **Vaginal colonization:** Is defined as the presence of GBS, *S. aureus*, *E. coli*, *Acinetobacter, Klebsiella* spp.*, Pseudomonas* spp.*, Proteus mirabilis*, *Citrobacter* spp., and *Candida* species isolated from vaginal swab cultures of pregnant women, regardless of symptoms.
- **Vertical transmission:** Is defined as the detection/isolation of the identical GBS, *S. aureus*, *E. coli*, *Acinetobacter, Klebsiella* spp.*, Pseudomonas* spp.*, Proteus mirabilis*, *Citrobacter* spp., and *Candida* spp. both in neonates and the mothers’ vaginal cultures obtained during pregnancy in cases of vaginal delivery.
- **Multidrug resistant (MDR):** isolates resistant to at least one antimicrobial in three or more antimicrobial categories.

### 2.7 Quality assurance

All quality control measures were conducted, starting with the development of a semi-structured questionnaire in the English language and translating it into the local language (Amharic). Then, it was translated back to the English language to assure consistency, followed by a pre-test on 5% of the total sample size (17 clients) at Kombolcha General Hospital, and modifications to questions were made accordingly. Additionally, half day training was given to the data collectors. Standard operating procedures and the manufacturer’s instructions were strictly followed during specimen collection, transportation, and processing steps. The collected data was checked daily for consistency and accuracy. A vaginal swab specimen not labeled, delayed beyond 24 h, or not transported at the appropriate temperature was rejected. The sterility of culture media was checked by incubating 5% of the media without inoculation (30). Moreover, visual inspections were applied to prepare and store media for holes, uneven filling, hemolysis, signs of freezing, bubbles, and corrosion.

The performance of the culture media was checked using Type Culture Collection (ATCC) standard reference strains such as *E. coli* (ATCC 25922), *E. faecalis* (ATCC 29212), *S. aureus* (ATCC 25923), *S. pyogenes* (ATCC 19615), *K. pneumoniae* (ATCC 700603), and *S. agalactiae* (ATCC 27956). The performance of THB was checked by inoculating the broth with known Gram-negative bacteria, *E. coli* (ATCC 25922) and *S. agalactiae* (ATCC 27956). The quality control of the disc diffusion technique was done by inoculating a quality control strain of *E. faecalis* (ATCC 29212) and a co-trimoxazole disc. The quality control of each biochemical test was checked by using different quality control strains for each test obtained from the Ethiopia Public Health Institute (EPHI). All reagents used for Gram stain were filtered before use, and quality control slides were used when performing Gram stain. The expiration date of all reagents, supplies, and antimicrobial discs was checked (30).

### 2.8 Data processing and statistical analysis

The data was coded and entered into Epi Data version 4.6.0 software and exported and analyzed by SPSS version 25.0 (IMB, USA). Descriptive statistics were computed to describe relevant variables and presented in tables. The reliability coefficient was checked using Cronbach’s alpha (*p* = 0.814). Bi-variable analysis was carried out to identify the possible associated factors with outcome variables. Variables with a *p*-value ≤0.25 were further entered into the multivariable logistic regression analysis. The Hosmer-Lemeshow goodness of fit test was computed, resulting in the model being fitted for analysis. Finally, variables with an adjusted odd ratio and a *p*-value less than 0.05 with a 95% CI were considered statistically significant.

### 2.9 Ethical considerations

The study was ethically approved by the Bahir Dar University College of Medicine and Health Science Ethical Review Board (Protocol number 749/2023). After describing the purpose of the study, DCSH and DHC granted a permission letter. Written informed consent from the pregnant mother were obtained before starting the study and assents from the newborns, following an explanation of the purpose, the benefits, and the possible risks of the study. To maintain confidentiality, personal identifiers were not utilized, and data were retrieved only for the purpose of the study. For each confirmed positive result, the responsible clinician of the participant was informed and treated based on the findings using an appropriate treatment protocol.

## 3 Results

### 3.1 Socio-demographic and clinical characteristics of pregnant women

A total of 348 pregnant women attending vaginal delivery were included in this study. More than half (54.9%) were below or equal to the age of 28, 263 (75.6%) were urban dwellers, 311 (89.4%) were married, 123 (35.3%) had a high-grade education level, 225 (64.7%) were housewives, 302 (86.8%) used public tap water as a source of drinking water, and 271 (77.9%) had no domestic animals in their house. Regarding the clinical characteristics, 21 (6.0%), 14 (4%), 10 (2.9%), and 48 (13.8%) pregnant women had a history of hospitalization in the past 3 months, were positive for HIV, positive for syphilis, and had a history of UTI during the current pregnancy, respectively (Table 1).

**Table 1:** Socio-demographic and clinical characteristics of pregnant women attending public health facilities in Dessie town, Northeast Ethiopia, 2023 (*N* = 348).

Variables	Clinical characteristics											
	History of hospitalization		HIV status		Syphilis status		History of UTI at current pregnancy		Vaginal discharge		History of STI	
	Yes	No	Positive	Negative	Positive	Negative	Yes	No	Yes	No	Yes	No
	*N* (%)	*N* (%)	*N* (%)	*N* (%)	*N* (%)	*N* (%)	*N* (%)	*N* (%)	*N* (%)	*N* (%)	*N* (%)	*N* (%)
Health facility												
DCSH	16 (4.6)	239 (68.7)	10 (2.9)	245 (70.4)	9 (2.6)	246 (70.7)	34 (9.8)	221 (63.5)	254 (73)	1 (0.3)	3 (0.9)	252 (72.4)
DHC	5 (1.4)	88 (25.3)	4 (1.1)	89 (25.6)	1 (0.3)	92 (26.4)	14 (4)	79 (22.7)	91 (26.2)	2 (0.5)	5 (1.4)	88 (25.3)
Total	21 (6.0)	327 (94.0)	14 (4.0)	334 (96.0)	10 (2.9)	338 (97.1)	48 (13.8)	300 (86.2)	345 (99.1)	3 (0.9)	8 (2.3)	340 (97.7)
Age (in years)												
≤28	13 (3.7)	178 (51.2)	8 (2.3)	183 (52.6)	5 (1.4)	186 (53.5)	22 (6.3)	26 (7.5)	189 (54.3)	2 (0.6)	2 (0.6)	189 (54.3)
>28	8 (2.3)	149 (42.8)	6 (1.7)	151 (43.4)	5 (1.4)	152 (43.7)	169 (48.6)	131 (37.6)	156 (44.8)	1 (0.3)	6 (1.7)	151 (43.4)
Residence												
Urban	16 (4.6)	247 (71)	12 (3.4)	251 (72.1)	4 (1.2)	259 (74.4)	36 (10.3)	227 (65.2)	260 (74.7)	3 (0.9)	8 (2.3)	255 (73.3)
Rural	5 (1.4)	80 (23)	2 (0.6)	83 (23.9)	6 (1.7)	79 (22.7)	12 (3.5)	73 (21)	85 (24.4)	0 (0)	0 (0)	85 (24.4)
Marital status												
Single	2 (0.6)	17 (4.9)	1 (0.3)	18 (5.2)	0 (0)	19 (5.5)	0 (0)	19 (5.5)	19 (5.5)	0 (0)	2 (0.6)	17 (4.9)
Married	19 (5.5)	292 (83.9)	12 (3.5)	299 (85.9)	10 (2.9)	301 (86.5)	46 (13.2)	265 (76.1)	308 (88.5)	3 (0.9)	5 (1.4)	306 (87.9)
Divorced	0 (0)	14 (4)	1 (0.3)	13 (3.7)	0 (0)	14 (4)	1 (0.3)	13 (3.7)	14 (4)	0 (0)	1 (0.3)	13 (3.8)
Widowed	0 (0)	4 (1.1)	0 (0)	4 (1.1)	0 (0)	4 (1.1)	1 (0.3)	3 (0.9)	4 (1.1)	0 (0)	0 (0)	4 (1.1)
Educational status												
Unable to read & write	3 (0.9)	24 (6.9)	1 (0.3)	26 (7.5)	1 (0.3)	26 (7.5)	7 (2)	20 (5.7)	27 (7.8)	0 (0)	1 (0.3)	26 (7.5)
Primary	5 (1.4)	90 (25.9)	6 (1.7)	89 (25.6)	5 (1.4)	90 (25.9)	13 (3.7)	82 (23.6)	95 (27.3)	0 (0)	1 (0.3)	94 (27)
Secondary	5 (1.4)	98 (28.2)	3 (0.9)	100 (28.7)	3 (0.9)	100 (28.7)	15 (4.3)	88 (25.3)	103 (29.6)	2 (0.6)	3 (0.9)	100 (28.7)
Diploma & above	8 (2.3)	115 (33)	4 (1.1)	119 (34.2)	1 (0.3)	122 (35)	13 (3.7)	110 (31.6)	123 (35.4)	1 (0.3)	3 (0.9)	120 (34.4)
Occupation												
Civil servant	4 (1.2)	36 (10.3)	3 (0.9)	37 (10.6)	0 (0)	40 (11.5)	7 (2)	33 (9.5)	39 (11.2)	1 (0.3)	2 (0.6)	38 (11)
Student	0 (0)	14 (4)	0 (0)	14 (4)	0 (0)	14 (4)	0 (0)	14 (4)	14 (4)	0 (0)	1 (0.3)	13 (3.7)
Farmer	2 (0.6)	16 (4.6)	0 (0)	18 (5.2)	1 (0.3)	17 (4.9)	4 (1.2)	14 (4)	18 (5.2)	0 (0)	1 (0.3)	17 (4.9)
House wife	11 (3.2)	214 (61.5)	8 (2.3)	217 (62.4)	8 (2.3)	217 (62.4)	29 (8.3)	196 (56.3)	224 (64.4)	1 (0.3)	4 (1.2)	221 (63.5)
Merchant	4 (1.2)	32 (9.1)	1 (0.3)	35 (10)	1 (0.3)	35 (10)	4 (1.2)	32 (9.2)	35 (10)	1 (0.3)	0 (0)	36 (10.3)
Daily labor	0 (0)	15 (4.3)	2 (0.6)	13 (3.7)	0 (0)	15 (4.3)	4 (1.2)	11 (3.2)	15 (4.3)	0 (0)	0 (0)	15 (4.3)
Source of drinking water												
Public tap water	17 (4.9)	285 (81.9)	12 (3.5)	290 (83.3)	8 (2.3)	294 (84.5)	38 (10.9)	264 (75.9)	300 (86.2)	2 (0.6)	7 (2)	295 (84.8)
Spring tap water	3 (0.9)	18 (5.1)	1 (0.3)	20 (5.7)	2 (0.6)	19 (5.5)	4 (1.2)	17 (4.9)	21 (6)	0 (0)	1 (0.3)	20 (5.7)
Private tap water	1 (0.3)	24 (6.9)	1 (0.3)	24 (6.9)	0 (0)	25 (7.1)	6 (1.7)	19 (5.4)	24 (6.9)	1 (0.3)	0 (0)	25 (7.2)
Domestic animal in the house												
Yes	3 (0.9)	74 (21.3)	3 (0.9)	74 (21.3)	3 (0.9)	74 (21.3)	12 (3.4)	65 (18.7)	77 (22.1)	0 (0)	0 (0)	77 (22.1)
No	18 (5.1)	253 (72.7)	11 (3.2)	260 (74.7)	7 (0.2)	264 (75.9)	36 (10.3)	235 (67.5)	268 (77)	3 (0.9)	8 (2.3)	268 (77)

DCSH, Dessie Comprehensive Specialized Hospital; DHC, Dessie Health Center; UTI, Urinary Tract Infection; STI, Sexually Transmitted Infection; and HIV, Human Immune Virus.

### 3.2 Obstetrics-related characteristics and vaginal colonization of pregnant women

Fifty (14.4%) of women delivered before 37 weeks of gestation; 29 (8.3%) and 19 (5.5%) had a history of abortion and stillbirth in their respective orders. Of the total pregnant women, 36 (10.3%) and 23 (6.6%) of them had experienced PROM and meconium-stained amniotic fluid during delivery, respectively. The percentage of potential neonatal pathogen colonization was higher among pregnant women who had: current gestational hyperbaton 8 (80%), history of stillbirth 12 (63.2%), membrane ruptures before the start of labor 33 (91.7%), and meconium-stained amniotic fluid 18 (78.3%) than their counterparts (Table 2).

**Table 2:** Obstetrics-related characteristics and colonization of potential neonatal pathogens among pregnant women in public health facilities of Dessie town, Northeast Ethiopia, 2023.

Variables	Category	Number of pregnant women *N* (%)	Mothers culture positives *N* (%)
Gestational age	<37	50 (14.4)	29 (58)
	≥37	298 (85.6)	164 (55)
Number of ANC visit	<4	115 (33)	62 (53.9)
	≥4	233 (67)	131 (56.2)
History of antibiotic use during the current pregnancy	Yes	18 (5.2)	10 (55.6)
	No	330 (94.8)	183 (55.5)
Current gestational hyperbaton	Yes	10 (2.9)	8 (80)
	No	338 (97.1)	185 (54.7)
Current gestational DM	Yes	4 (11)	2 (50)
	No	344 (98.9)	191 (55.5)
Type of gravidia	Primigravida	116 (33.3)	57 (49.1)
	Multigravida	232 (66.7)	136 (58.6)
History of abortion	Yes	29 (8.3)	14 (48.3)
	No	319 (91.7)	179 (56.1)
History of still birth	Yes	19 (5.5)	12 (63.2)
	No	329 (94.5)	181 (55)
PROM	Yes	36 (10.3)	33 (91.7)
	No	312 (89.7)	160 (37.2)
Duration of labor (in hour)	≤12	215 (61.8)	77 (35.8)
	>12	133 (38.2)	69 (51.3)
Fever during labor	Yes	43 (12.4)	21 (48.8)
	No	305 (87.6)	172 (56.4)
Meconium-stained amniotic fluid	Yes	23 (6.6)	18 (78.3)
	No	325 (93.4)	175 (53.9)

ANC, Anti-natal care; DM, Diabetes mellitus; PROM, Premature rupture of membrane.

### 3.3 Socio-demographic characteristics and colonization of potential neonatal pathogens among newborns

Of the 348 newborns, 182 (52.3%) were female, and 56 (16.1%) had low birth weight (≤2,500 g). The percentage of potential neonatal pathogen colonization was higher among: those with <2.5 kg birth weights (43, 76.8%), and 13 (41.9%) had an APGAR score of <7 than their counterparts (Table 3).

**Table 3:** Socio-demographic characteristics and colonization of potential neonatal pathogens among newborns in public health facilities in Dessie town, Northeast Ethiopia, 2023.

Variable	Number of newborn *N* (%)	Newborn culture positives *N* (%)
Sex of newborn		
Male	166 (47.7)	57 (34.3)
Female	182 (52.3)	47 (25.8)
Weight of newborns (Grams)		
<2,500	56 (16.1)	43 (76.8)
≥2,500	292 (83.9)	61 (20.9)
5th minute APGAR score		
<7	31 (8.9)	13 (41.9)
7–10	317 (91.1)	91 (28.7)
Status of newborn at birth		
Alive	346 (99.4)	103 (29.8)
Dead	2 (0.6)	1 (50)

APGAR, appearance, pulse, grimace, activity, respiration.

### 3.4 Vaginal colonization in pregnant women and vertical transmission to newborns

The magnitude of maternal and neonatal colonization with at least one potential neonatal pathogen was 55.5% (*n* = 193; 95% CI: 50.5, 60.4%) and 29.9% (*n* = 104; 95% CI: 25.3, 34.8%), respectively. Among the colonized women and newborns, 31 and 10, respectively, were colonized with more than one potential neonatal pathogen. The most frequently isolated potential pathogens among pregnant women in labor were *E. coli* 96 (27.9%), *Candida* spp. 49 (14.1%), *Klebsiella* spp. 21 (6%), and *S. aureus* 17 (4.9%). On the contrary, *E. coli* 56 (16.1%), *Candida* spp. 22 (6.3%), *Klebsiella* spp. 12 (3.4%), and *S. aureus* 7 (2%) were the most frequently isolated potential pathogens among newborns. The overall proportion of vertical transmission of potential neonatal pathogens was 53.9% (104/193, 95% CI: 45.5, 61.7). The highest vertically transmitted pathogens were *E. coli* (57.7%), followed by *Klebsiella* spp. (57.1%), *Acinetobacter* spp. (54.6%), *Candida* spp. (44.9%), *S. aureus* (41.2%), and GBS (41.7%) (Table 4).

**Table 4:** Potential neonatal pathogen colonization profile of pregnant women and newborns and rate of vertical transmission in public health facilities in Dessie town, Northeast Ethiopia, 2023.

Pathogens	Culture positive pregnant mothers *N* = 348; No (%)	Culture positive newborn *N* = 348; No (%)	Proportion of vertical transmission; %	Pearson correlation coefficient (*r*)	*p*-value
*E. coli*	97 (27.9)	56 (16.1)	57.7	0.71	<0.001
*Candida* spp.*	49 (14.1)	22 (6.3)	44.9	0.64	<0.001
*Klebsiella* spp.**	21 (6)	12 (3.4)	57.1	0.75	<0.001
*S. aureus*	17 (4.9)	7 (2)	41.2	0.63	<0.001
GBS	12 (3.4)	5 (1.4)	41.7	0.69	<0.001
*Acinetobacter* spp.	11 (3.2)	6 (1.7)	54.5	0.73	<0.001
*P. mirabilus*	6 (1.7)	2 (0.6)	33.3	0.57	<0.001
*Citrobactor* spp.	6 (1.7)	2 (0.6)	33.3	0.57	<0.001
*Pseudomonas* spp.	6 (1.7)	2 (0.6)	33.3	0.31	<0.001
Total	225	114	50.7	0.59	<0.001

*Candida albicans = 31, *Candida krusei* = 18, **K. pneumoniae = 13, *K. oxytoca* = 8.

The Pearson correlation coefficient (*r*) showed a positive association between maternal colonization and vertical transmission rate. Generally, the proportion of vertical transmission had a stronger association with vaginal colonization of potential pathogens (*r* = 0.59, *p* < 0.001). Regarding species distribution and their correlation, the vaginal colonization and vertical transmission of potential pathogens had a strong correlation with *r* > 0.50 and *p* < 0.001 except for *Pseudomonas* spp., where there was a moderate association between vaginal colonization and vertical transmission (*r* = 0.31, *p* < 0.001) (Table 4).

### 3.5 Antimicrobial susceptibility profile of bacterial isolates

Antimicrobial susceptibility testing was performed on 244 bacterial isolates for five selected genera in the current study, namely GBS (*n* = 17, 7%), *S. aureus* (*n* = 24, 9.8%), *E. coli* (*n* = 153, 62.7%), *Klebsiella* spp. (*n* = 33, 13.5%), and *Acinetobacter* spp. (*n* = 17, 7%). Tetracycline, erythromycin, and clindamycin resistance against GBS isolates was present in 14 (82.4%), 9 (52.9%), and 6 (35.3%), respectively. Of the 153 *E. coli* isolates, 143 (94.5%) were resistant to ampicillin, 89 (58.2%) to cefazolin, 78 (51.0%) to ciprofloxacin, and 0 (0%) to amikacin. Furthermore, the level of resistance of *Klebsiella* species against cefazolin and amikacin was 28 (84.9%) and 0 (0%), respectively. Similarly, resistance to ceftriaxone 13 (76.5%), trimethoprim-sulfamethoxazole 5 (29.4%), and imipenem 5 (29.4%) was demonstrated by *Acinetobacter* spp. (Table 5).

**Table 5:** Antimicrobial susceptibility patterns of potential neonatal pathogen in public health facilities of Dessie town, Northeast Ethiopia, 2023.

Antibiotics	Isolates, number (%)									
	GBS (*n* = 17)		*S. aureus* (*n* = 24)		*E. coli* (*n* = 153)		*Klebsiella* spp. (*n* = 33)		*Acinetobacter* spp. (*n* = 17)	
	S	R	S	R	S	R	S	R	S	R
Ampicillin	15 (88.2)	2 (11.8)	–	–	10 (5.5)	143 (94.5)	–	–	–	–
Clindamycin	11 (64.7)	6 (35.3)	18 (75)	6 (25)	–	–	–	–	–	–
Erythromycin	8 (47.1)	9 (52.9)	11 (45.8)	13 (54.2)	–	–	–	–	–	–
Tetracycline	3 (17.6)	14 (82.4)	13 (54.2)	11 (45.8)	–	–	–	–	–	–
Cefoxitin	–	–	19 (79.2)	5 (20.8)	–	–	–	–	–	–
Penicillin	13 (76.5)	4 (23.5)	21 (87.5)	3 (12.5)	–	–	–	–	–	–
Chloramphenicol	12 (70.6)	5 (29.4)	22 (91.7)	2 (8.3)	–	–	–	–	–	–
Ciprofloxacin	–	–	13 (54.2)	11 (45.8)	75 (49.0)	78 (51.0)	14 (42.4)	19 (57.6)	10 (58.8)	7 (41.2)
Trimethoprim-Sulfamethoxazole	–	–	12 (50)	12 (50)	119 (77.7)	34 (22.3)	26 (78.8)	7 (21.2)	12 (70.6)	5 (29.4)
Ceftriaxone	14 (82.4)	3 (17.6)	–	–	85 (55.6)	68 (44.4)	18 (54.5)	15 (44.5)	4 (23.5)	13 (76.5)
Gentamicin	–	–	18 (75)	6 (25)	103 (67.3)	50 (32.7)	20 (60.7)	13 (30.3)	12 (70.6)	5 (29.4)
Amoxicillin -clavulanate	–	–	–	–	139 (90.9)	14 (9.1)	29 (87.9)	4 (12.1)	–	–
Amikacin	–	–	–	–	153 (100)	0 (0)	33 (100)	0 (0)	11 (64.7)	6 (36.3)
Imipenem	–	–	–	–	106 (69.3)	47 (30.7)	26 (78.8)	7 (21.2)	12 (70.6)	5 (29.4)
Meropenem	–	–	–	–	111 (72.5)	42 (27.5)	13 (39.3)	20 (60.7)	11 (64.7)	6 (35.3)
Ceftazidime	–	–	–	–	134 (87.6)	19 (12.4)	26 (78.8)	7 (21.2)	11 (64.7)	6 (35.3)
Aztreonam	–	–	–	–	139 (90.8)	14 (9.2)	32 (97)	1 (3.0)	–	–
Cefazolin	–	–	–	–	64 (41.8)	89 (58.2)	5 (15.1)	28 (84.9)	–	–

R, Resistant; S, Sensitive.

#### 3.5.1 Multi-drug resistance patterns of bacterial isolates

Out of the total 244 isolates, 91 (37.3%) were MDR (showing resistance to three or more antibiotics from different classes). Two pairs of GBS, *S. aureus* and *Acinetobacter* spp. isolates exhibited similar multi-drug resistance pattern. Additionally, 22 pairs of *E. coli* and 6 pairs of *Klebsiella* spp. were MDR pathogens (Table 6).

**Table 6:** Multi-drug resistance profile of potential neonatal pathogen isolates identified from pregnant women and their newborns in public health facilities in Dessie town, Northeast Ethiopia, 2023.

Isolated bacteria	Source	Degree of resistance						Total MDR
		R0	R1	R2	R3	R4	≥R5	
GBS	Maternal (12)	1 (8.3)	7 (58.3)	2 (16.7)	1 (8.3)	1 (8.3)	0 (0)	2 (16.7)
	Newborn (5)	1 (20.0)	1 (20.0)	1 (20.0)	1 (20.0)	1 (20.0)	0 (0)	2 (40)
*S. aureus*	Maternal (17)	6 (35.3)	5 (29.4)	3 (17.6)	3 (17.6)	0 (0)	0 (0)	3 (17.6)
	Newborn (7)	2 (28.6)	1 (14.3)	2 (28.6)	2 (28.6)	0 (0)	0 (0)	2 (28.6)
*E. coli*	Maternal (97)	2 (2.1)	41 (42.3)	17 (17.5)	14 (14.4)	7 (7.2)	17 (17.5)	38 (39.2)
	Newborn (56)	0 (0)	23 (41.1)	11 (19.6)	12 (21.4)	6 (10.7)	4 (7.1)	22 (39.3)
*Klebsiella* spp.	Maternal (21)	0 (0)	4 (19.0)	7 (33.3)	5 (23.8)	2 (9.5)	3 (14.3)	10 (47.6)
	Newborn (12)	0 (0)	2 (16.7)	4 (33.3)	3 (25)	2 (16.7)	1 (8.3)	6 (50.0)
*Acinetobacter* spp.	Maternal (11)	2 (18.2)	5 (45.5)	0 (0)	2 (18.2)	0 (0)	2 (18.2)	4 (36.4)
	Newborn (6)	1 (16.7)	3 (50.0)	0 (0)	1 (16.7)	0 (0)	1 (16.7)	2 (33.3)
Total	Maternal (158)	10 (6.3)	62 (39.2)	29 (18.4)	25 (15.8)	10 (6.3)	22 (13.9)	57 (36.1)
	Newborn (86)	4 (4.7)	30 (34.9)	18 (20.9)	19 (22.1)	9 (10.5)	6 (6.9)	34 (39.5)
Overall (244)		14 (5.7)	92 (37.7)	47 (19.3)	44 (18)	19 (7.8)	28 (11.5)	91 (37.3)

*n, Number of tested strains; R0, no resistance; R1, resistant to one class of antibiotics; R2, resistant to two class of antibiotics; R3, resistant to three class of antibiotics; R4, resistant to four class of antibiotics; ≥R5, resistant to five and above class of antibiotics; MDR, Multi Drug Resistance.*

### 3.6 Factors associated with vaginal colonization

In bivariate analysis, fever during labor, meconium-stained amniotic fluid, history of UTI at current pregnancy, domestic animal in the house, and PROM were associated with vaginal colonization of potential neonatal pathogens (*p* < 0.05). However, in the multivariable analysis, most of the variables tested in the regression model did not show a statistical association with potential neonatal pathogen colonization among pregnant women, including fever during labor and meconium-stained amniotic fluid (*p* > 0.05). However, domestic animals in the house (AOR: 2.88, 95% CI: 1.55–5.36, *p* = 0.001), PROM (AOR: 2.95, 95% CI: 1.29–6.74, *p* = 0.010), and current UTI (AOR: 2.66, 95% CI: 1.23–5.76, *p* = 0.013) were statistically significant with maternal potential neonatal pathogen colonization (Table 7).

**Table 7:** Factors associated with vaginal potential neonatal pathogen colonization in public health facilities of Dessie town, Northeast Ethiopia, 2023.

Factors		Pathogen colonization *n* (%)		COR (95% CI)	*p*-value	AOR (95% CI)	*p*-value
		Yes	No				
Age (Year)	≤28	101 (52.9)	90 (47.1)	1			
	>28	92 (58.6)	65 (41.4)	1.26 (0.82–1.93)	0.286		
Residence	Urban	145 (55.1)	118 (44.9)	1			
	Rural	48 (56.5)	37 (43.5)	1.06 (0.65–1.73)	0.829		
Marital status	Single	8 (42.1)	11 (57.9)	1			
	Married	174 (55.9)	137 (44.1)	1.75 (0.68–4.46)	0.244		
	Divorced	8 (57.1)	6 (42.9)	1.83 (0.45–7.41)	0.395		
	Widowed	3 (75)	1 (25)	4.13 (0.36–47.30)	0.255		
Educational status	Unable to read and write	15 (55.6)	12 (44.4)	0.92 (0.40–2.12)	0.837	0.51 (0.17–1.52)	0.224
	Elementary	46 (48.4)	49 (51.6)	0.69 (0.40–1.18)	0.173	0.49 (0.24–1.97)	0.410
	Secondary	61 (59.2)	42 (40.8)	1.06 (0.63–1.81)	0.820	0.99 (0.54–1.85)	0.993
	High grade	71 (57.7)	52 (42.3)	1		1	
Occupation	Civil servant	22 (55)	18 (45)	1		1	
	Student	6 (42.9)	8 (57.1)	0.61 (0.18–2.10)	0.436	0.68 (0.18–2.57)	0.570
	Farmer	9 (50)	9 (50)	0.82 (0.27–2.49)	0.724	0.64 (0.16–2.58)	0.526
	House wife	122 (54.2)	103 (45.8)	0.97 (0.49–1.91)	0.927	1.25 (0.55–2.81)	0.593
	Merchant	25 (69.4)	11 (30.6)	1.86 (0.72–4.78)	0.198	2.19 (0.81–5.93)	0.121
	Daily labor	9 (60)	6 (40)	1.23 (0.37–4.10)	0.739	1.45 (0.40–5.23)	0.574
Source of drinking water	Public tap water	167 (55.3)	135 (44.7)	1.14 (0.51–2.58)	0.750		
	Spring tape water	13 (61.9)	8 (38.1)	1.50 (0.46–4.88)	0.500		
	Private	13 (52)	12 (48)	1			
Domestic animal in the house	Yes	53 (68.8)	24 (31.2)	2.07 (1.21–3.54)	0.008*	2.88 (1.55–5.36)	0.001*
	No	140 (51.7)	131 (48.3)	1		1	
Gestational age (week)	<37	29 (58)	21 (42)	1.13 (0.62–2.07)	0.696		
	≥37	164 (55)	134 (45)	1			
Number of ANC visit	<4	62 (53.9)	53 (46.1)	0.91 (0.58–1.43)	0.683		
	≥4	131 (56.2)	102 (43.8)	1			
History of antibiotic use	Yes	10 (55.6)	8 (44.4)	1.00 (0.39–2.61)	0.993		
	No	183 (55.5)	147 (44.5)	1			
Type of gravidia	Primigravida	57 (52.8)	51 (47.2)	1			
	Multigravida	136 (56.7)	104 (43.3)	1.17 (0.74–1.85)	0.500		
History of abortion	Yes	14 (58.3)	10 (41.7)	1.13 (0.49–2.63)	0.769		
	No	179 (55.2)	145 (44.8)	1			
History of still birth	Yes	12 (75)	4 (25)	2.50 (0.79–7.92)	0.119	1.97 (0.57–6.84)	0.287
	No	181 (54.5)	151 (45.5)	1		1	
PROM	Yes	33 (76.7)	10 (23.3)	2.99 (1.42–6.28)	0.004	2.95 (1.29–6.74)	0.010*
	No	160 (52.5)	145 (47.5)	1		1	
Duration of labor (hour)	≤12	102 (50.5)	100 (49.5)	1		1	
	>12	91 (62.3)	55 (37.7)	0.62 (0.40–0.95)	0.029	1.41 (0.88–2.23)	0.154
Fever during labor	Yes	21 (48.8)	22 (51.2)	0.74 (0.39–1.40)	0.352	1	
	No	172 (56.4)	133 (43.6)	1			
Meconium-stained amniotic fluid	Yes	18 (78.3)	5 (21.7)	3.09 (1.12–8.51)	0.029	1.91 (0.63–5.77)	0.250
	No	175 (53.9)	150 (46.1)	1		1	
History of hospitalization in the past 3 months	Yes	12 (57.1)	9 (42.9)	1.08 (0.44–2.62)	0.873		
	No	181 (55.4)	146 (44.6)	1			
HIV status	Positive	10 (71.4)	4 (28.6)	2.06 (0.63–6.71)	0.229		
	Negative	183 (54.8)	151 (45.2)	1			
Syphilis status	Reactive	4 (40)	6 (60)	0.53 (0.15–1.90)	0.326		
	Non-reactive	189 (55.9)	149 (44.1)	1			
History of UTI at current pregnancy	Yes	37 (77.1)	11 (22.9)	3.11 (1.53–6.32)	0.002	2.66 (1.23–5.76)	0.013*
	No	156 (52)	144 (48)	1		1	
Presence vaginal discharge	Yes	1 (33.3)	2 (66.7)	0.40 (0.04–4.44)	0.454		
	No	192 (55.7)	153 (44.3)	1			
History of STI	Yes	5 (62.8)	3 (37.5)	1.35 (0.32–5.73)	0.686		
	No	1,529 (44.7)	188 (55.3)	1			

ANC, Anti Natal Care; DCSH, Dessie Comprehensive Specialized Hospital; DHC, Dessie Health Center; DM, Diabetes Miletus; HIV, Human Immune Virus; PROM, Premature rapture of membrane; STI, Sexually Transmitted Disease; UTI, Urinary Tract infection, (*), significant at *p*-value < 0.05; 1, reference category; AOR, Adjusted Odd Ratio; COR, Crude Odds Ratio; CL, confidence interval.

### 3.7 Factors associated with vertical transmission

Among the several factors assessed in the analysis of factors associated with vertical transmission of potential neonatal pathogens to newborns, there was a statistically significant association with duration of labor (AOR = 2.23, 95% CI = 1.11, 4.47, *p* = 0.024) and weight of newborns (grams) (AOR = 5.38, 95% CI = 2.17, 13.36, *p* ≤ 0.001). The likelihood of potential neonatal pathogen vertical transmission to newborns was 2.23 times higher for those newborns delivered by a pregnant mother whose duration of labor was >12 h than for those whose duration of delivery was ≤12 h. On the other hand, the odds of potential neonatal pathogen vertical transmission were 5.38 times higher for those newborns weighing less than 2,500 g when compared to their counterparts (Table 8).

**Table 8:** Factors associated with vertical transmission of potential neonatal pathogens to the newborns in public health facilities in Dessie town, Northeast Ethiopia, 2023 (*n* = 193).

Factors		Vertical transmission		COR (95% CI)	*p*-value	AOR (95% CI)	*p*-value
		Yes	No				
Age (Year)	≤28	49 (48.5)	52 (51.5)	1			
	>28	55 (59.8)	37 (40.2)	1.58 (0.89–2.79)	0.118	1.03 (0.51–2.09)	0.936
Residence	Urban	75 (51.7)	70 (48.3)	1		1	
	Rural	29 (60.4)	19 (39.6)	1.43 (0.73–2.77)	0.296		
Marital status	Single	3 (37.5)	5 (62.5)	1			
	Married	99 (56.9)	75 (43.1)	2.20 (0.51–9.50)	0.291		
	Divorced	1 (12.5)	7 (87.5)	0.24 (0.02–3.01)	0.268		
	Widowed	1 (33.3)	2 (66.7)	0.83 (0.05–13.63)	0.898		
Educational status	Unable to read and write	13 (86.7)	2 (13.3)	5.04 (1.06–23.99)	0.042	2.82 (0.47–16.83)	0.256
	Elementary	22 (47.8)	24 (52.2)	0.71 (0.34–1.50)	0.368	0.84 (0.34–2.04)	0.694
	Secondary	29 (47.5)	32 (52.5)	0.70 (0.35–1.40)	0.314	0.79 (0.35–1.77)	0.559
	High grade	40 (56.3)	31 (43.7)	1		1	
Occupation	Civil servant	13 (69.1)	9 (40.9)	1			
	Student	2 (33.3)	4 (66.7)	0.35 (0.05–2.31)	0.273		
	Farmer	5 (55.6)	4 (44.4)	0.87 (0.18–4.14)	0.856		
	House wife	65 (53.3)	57 (46.7)	0.79 (0.31–1.98)	0.615		
	Merchant	15 (41.7)	21 (58.3)	1.04 (0.32–3.34)	0.949		
	Daily labor	4 (26.7)	11 (73.3)	0.55 (0.12–2.65)	0.459		
Source of drinking water	Public tap water	92 (55.1)	75 (44.9)	2.76 (0.82–9.32)	0.102	2.03 (0.53–7.83)	0.302
	Spring tape water	8 (61.5)	5 (38.5)	3.60 (0.71–18.25)	0.122	3.19 (0.49–20.95)	0.228
	Private	4 (30.8)	9 (69.2)	1			
Domestic animal in the house	Yes	32 (60.4)	21 (39.6)	1.44 (0.76–2.74)	0.267		
	No	72 (51.4)	68 (48.6)	1			
Gestational age (Week)	<37	22 (75.9)	7 (24.1)	3.14 (1.27–7.76)	0.013	2.06 (0.72–5.89)	0.176
	≥37	82 (50)	82 (50)	1		1	
Number of ANC visit	<4	31 (50)	31 (50)	0.80 (0.43–1.46)	0.457		
	≥4	73 (55.7)	58 (44.3)	1			
History of antibiotic use	Yes	5 (50)	5 (50)	1.18 (0.33–4.21)	0.800		
	No	99 (54.1)	84 (45.9)	1			
Type of gravidia	Premigravidia	28 (49.1)	29 (50.9)	1			
	Multigravidia	76 (55.9)	60 (44.1)	1.31 (0.71–2.44)	0.391		
History of abortion	Yes	10 (71.4)	4 (28.6)	2.26 (0.68–7.48)	0.181	1.40 (0.35–5.56)	0.663
	No	94 (52.5)	85 (47.5)	1		1	
History of still birth	Yes	4 (33.3)	8 (66.7)	0.41 (0.12–1.39)	0.152	0.36 (0.08–1.68)	0.192
	No	100 (55.2)	81 (44.8)	1		1	
PROM	Yes	19 (57.6)	14 (42.4)	1.20 (0.56–2.55)	0.641		
	No	85 (53.1)	75 (46.9)	1			
Duration of labor (Hour)	≤12	42 (41.2)	60 (58.8)	1		1	
	>12	62 (68.1)	29 (31.9)	3.05 (1.69–5.52)	0.000	2.23 (1.11–4.47)	0.024*
Fever during labor	Yes	8 (38.1)	13 (61.9)	0.49 (0.19–1.24)	0.130	0.33 (0.10–1.12)	0.076
	No	96 (55.8)	76 (44.2)	1		1	
Meconium stained amniotic fluid	Yes	9 (50)	9 (50)	0.84 (0.32–2.22)	0.729		
	No	95 (54.3)	80 (45.7)	1			
History of hospitalization in the past 3 months	Yes	4 (33.3)	8 (66.7)	0.41 (0.12–1.39)	0.152	0.60 (0.13–2.72)	0.511
	No	100 (55.2)	81 (44.8)	1		1	
HIV status	Positive	4 (40)	6 (60)	0.55 (0.15–2.03)	0.372		
	Negative	100 (54.6)	83 (45.4)	1			
Syphilis status	Reactive	3 (75)	1 (25)	2.61 (0.27–25.58)	0.409		
	Non-reactive	101 (53.4)	88 (46.6)	1			
History of UTI at current pregnancy	Yes	24 (64.9)	13 (35.1)	1.75 (0.83–3.69)	0.139	1.38 (0.54–3.55)	0.506
	No	80 (51.3)	76 (48.7)	1		1	
History of STI	Yes	3 (60)	2 (40)	1.29 (0.21–7.99)	0.782		
	No	101 (53.7)	87 (46.3)	1			
Sex of newborn	Male	57 (58.8)	40 (41.2)	1		1	
	Female	47 (49)	49 (51)	0.67 (0.38–1.19)	0.173	1.53 (0.78–2.99)	0.212
Weight of newborns (Gram)	<2,500	43 (82.7)	9 (17.3)	6.27 (2.84–13.83)	0.000	5.38 (2.17–13.36)	<0.001*
	≥2,500	61 (43.3)	80 (56.7)	1		1	
5th minute APGAR score	<7	13 (54.2)	11 (45.8)	1.01 (0.43–2.39)	0.976		
	7–10	91 (53.8)	78 (46.2)	1			

ANC, Anti Natal Care; DCSH, Dessie Comprehensive Specialized Hospital; DHC, Dessie Health Center; DM, Diabetes Miletus; HIV, Human Immune Virus; PROM, Premature rapture of membrane; STI, Sexually Transmitted Disease; UTI, Urinary Tract Infection; (*), significant at *p*-value < 0.05; 1, reference category; AOR, Adjusted Odd Ratio; COR, Crude Odds Ratio; CL, confidence interval.

## 4 Discussion

The newborn’s health may be impacted by specific bacterial species that colonize the vagina in the later stages of pregnancy. Before intrapartum antimitotic prophylaxis was used to treat newborn illness, *S. agalactiae* was the main cause of the condition in industrialized countries. Ethiopia and other impoverished countries, however, do not frequently report invasive *S. agalactiae* infections. Instead of *S. agalactiae*, other bacteria are commonly recovered from neonates with sepsis. According to recent research, pathogens found in the blood of infants with early-onset sepsis have been linked to mother vaginal colonization with several pathogenic bacteria (31).

In this study, 55.5% of the women had vaginal colonization with potential neonatal pathogens. The findings of the present study agreed with findings reported by 58.4% in Sri Lanka (32) and 52.6% in India (33). However, it was higher than 34.7 and 37.2%, respectively, in Hawassa, Ethiopia (19), and Bangladesh (34) studies. On the other hand, our finding was lower than the prevalence reported from Central Uganda (64.9%) (23) and Indonesia (62.2%) (16). The contradictory results may also be attributed to population characteristics such as antibiotic use, genetics, nutritional status, and other characteristics among the people under study. Additionally, it is also dependent on the type of microbiological methods. If an enrichment broth and rectal swabs are used, the carriage rate would be change to higher rates. Furthermore, socioeconomic and environmental factors related to the study areas might affect how women practice personal hygiene and are exposed to infections. Developing nations frequently have worse sanitation, which may have an effect on colonization rates.

According to the present study, *E. coli*, *Candida* spp., and *Klebsiella* spp. were the most common isolated pathogens, which was supported by the previous findings reported from central Uganda (23), Sri Lanka (32), and Ethiopia (19). Moreover, *E. coli* was the predominant pathogen isolated from the recto-vaginal culture of the pregnant mother in a study in Iran (35).

The vaginal colonization of *E. coli* among women was 27.6% (95% CI: 22.4, 32.6%), which was concurrent with previous studies conducted in Ethiopia (29.9%) (19) and Iran (29.34%) (35) while it was higher than findings reported from Bangladesh (11.1%) (34), Sri Lanka (5.6%) (32), USA (12%) (36), Spain (13%) (37), India (8.9%) (33), and Korea (1.7%) (38). Variations in the sociodemographic characteristics of study participants, behaviors, and practices toward personal hygiene might contribute to the aggravating magnitude of *E. coli* in the present study compared to the USA and other developed countries.

On the contrary, the *E. coli* colonization rate was lower compared with reports from South Africa (33.3%) (39), Central Uganda (49.6%) (23), India (38.8%) (40), and Iran (55.4%) (41). Variations in the methodological approach, sample size, sanitary settings, environmental factors, and the type of participants may contribute to differences in the colonization rate. For example; all the Indian participants were pregnant mothers complaining of vaginal discharge; in the Iranian study, samples were taken from recto-vaginal areas; and Ugandan participants were pregnant women with obstructed labor, which may increase the possibility of higher colonization.

*Klebsiella* spp. was the second most frequently identified vaginal colonizing pathogen with a colonization rate of 6% (95% CI: 3.9, 9.1%), which was higher than reports from Ethiopia (1.5%) (19), India (2%) (33), Iran (0.25%) (35), and Korea (0.4%) (38). In the present study, the species of *Klebsiella* were not identified, unlike previous studies from Ethiopia, where the prevalence of these studies was determined among *K. pneumoniae*. This might contribute to the higher prevalence in the present study. Additionally, in a Morocco study, the API 10S system was used to confirm *Klebsiella* colonies, which had better specificity than routine culture. Furthermore, *Candida* spp. was the other predominant vaginal colonizing pathogen next to *Klebsiella* spp., with a colonization rate of 14.1% (95% CI: 10.8, 19.1%), which was higher than studies conducted in Iran (9.8%) (35). Despite the fact that we identified the species of *Candida*, the proportion was determined from the total species, which is unlikely to match the findings of Iran, where the colonization rate was calculated from *C. albicans* only. This methodological approach to determining the proportion might be the reason for the discrepancy.

In this study, 29.9% (104/348; 95% CI 25.5, 34.3%) of newborns were colonized by at least one potential neonatal pathogen. The predominant neonatal colonizing pathogen was *E. coli,* followed by *Candida* spp., and *Klebsiella* spp. This finding was supported by the previous finding from Iran (35). Moreover, the predominancy of these species is quite similar to vaginal colonization. Thus, the predominance might be due to the fact that *E. coli* is a member of the normal microbiota found in the gastrointestinal tract, which is associated with severe infections and meningitis in neonates. Following a normal vaginal birth, newborns can be frequently colonized with maternal vaginal and fecal flora; hence, newborns can acquire it during or before delivery (42).

The overall magnitude of vertical transmission of potential neonatal pathogens was 53.9% (104/193, 95% CI: 45.5, 61.7). The magnitude of vertical transmission implies that among the two pregnant women who had vaginal colonization of a potential neonatal pathogen, vertical transmission will occur from one woman to her newborn. This finding implies that improving the health of the mother leads to reduce the rate of vertical transmission. Moreover, improving infection prevention practices will improve the MCH by reducing hospital acquired infections. The other implication of this finding was the establishment and improvement of diagnostic facilities and/or microbiology laboratories. Furthermore, there was a strong positive association between maternal vaginal colonization and overall vertical transmission of potential neonatal pathogens (*r* = 0.59, *p* < 0.001). A similar strong positive association (*r* > 0.5, *p* < 0.001) was observed between vaginal colonization and vertical transmission of each species unlikely to *Pseudomonas* spp. (*r* = 0.31, *p* < 0.001), where it had a moderate positive association.

The frequency of vertical transmission rates of *E. coli*, *Klebsiella* spp., *Acinetobacter* spp., *Candida* spp., GBS, and *S. aureus* was 59.4, 57.1, 54.6, 44.9, and 41.7%, 41.2%, respectively. The frequency of the first two pathogens, i.e., *E. coli*, and *Klebsiella* spp., was supported by previous findings from Iran (35). Moreover, this study showed that the first three pathogens had a vertical transmission likelihood of more than 50%. Unfortunately, these pathogens are the predominant cause of EOS in low- and middle-income countries (10, 43), including Ethiopia, where *E. coli* and *K. pneumoniae* were the predominant etiological agents of EOS (12–15).

The highest frequency of *E. coli* isolates showing resistance to ampicillin, cefazolin, and ciprofloxacin was 93.5, 57.4, and 51%, respectively. However, it had better sensitivity for amikacin, amoxicillin-clavulanic acid, and ceftazidime at 100, 90.9, and 89.2%, respectively. The level of ampicillin resistance was alarmingly higher, which was supported by previous results in Central Uganda of 89.4% (23) but higher than 62.1% in Iran (44). The observed differences between these studies may be due to variations in drug control policies, the number of isolates tested, and community awareness of medication resistance. However, the extensive empirical use of these antibiotics in the management of various infectious diseases might speed up the rise of antibiotic-resistant bacteria. The World Health Organization suggests ampicillin as the initial empirical treatment option for newborn sepsis (45). Similarly, the level of resistance of *Klebsiella* spp. isolates to cefazolin, ciprofloxacin, and ceftriaxone was 84.7, 57.6, and 45.5%, respectively. This study revealed that ceftriaxone is still an effective antibiotic against *Klebsiella* spp. This high level of resistance was in agreement with previous findings (46). Furthermore, *Acinetobacter* spp. isolates showed 76.5% resistance against ceftriaxone, which was consistent with 80% resistance in India (41). This implies ceftriaxone is no longer used as a treatment for pregnant mothers and newborns colonized with *Acinetobacter* spp. in the area.

GBS isolates showed 62.9% resistance to erythromycin, which was higher than evidence from Bahirdar (25.9%) (47), Jigjiga (20.7%) (48), Cameron (49%) (49), and Uganda (21%) (50). Variations in the degree of resistance may result from the study participants’ different cultural backgrounds, socioeconomic backgrounds, and geographic locations. Moreover, the level of resistance of GBS isolates to tetracycline was 82.4%, which was concurrent with a study in Bahirdar (87%) (47). This finding implies that tetracycline is no longer an effective antibiotic for treating or preventing GBS isolates, as evidenced by the significant proportion of resistant isolates. Chloramphenicol was found to have a better sensitivity to GBS (91.7%), which was consistent with previous Ethiopian studies such as Bahirdar (87%) (47) and Arbaminch (88.6%) (51). Generally, a significant level of resistance to these antibiotics might be due to the unregulated extended use of these antibiotics for various infections.

According to the international standards for drug resistance definition, it was found that 37.3% of the isolates had multidrug resistance. This finding was consistent with the finding reported from Bahir Dar, Ethiopia (48). This high burden of multidrug resistant bacteria is alarming, as they are already resistant to several first-line antibiotics that could potentially spread from mothers to newborns (52). Infection or colonization with multidrug resistant bacteria reduces treatment options and raises healthcare expenses, morbidity, and mortality (53). Additionally, these pathogens can proliferate and spread in clinical environments, offering a continuous risk. To stop the spread of MDR, more surveillance must be conducted in addition to the best infection control and antimicrobial stewardship procedures being put into place (54). The high level of antimicrobial resistance (AMR) observed in our study may be attributed to various factors, such as the overuse and abuse of drugs in the study area, which has lax drug regulation practices and a sparse bacteriological surveillance system due to the absence of routine anticoagulant testing. The majority of the above-mentioned antimicrobials are sold locally, and most people take them without a prescription.

The most predominant MDR pathogen in this study was *E. coli* (39.2%), followed by *Acinetobacter* spp. (35.3%). The predominance of MDR *E. coli* might be due to the synthesis of extended spectrum beta-lactamase (ESBL) and carbapenemases enzymes, the loss of porins, which will further inhibit the permeability of drugs, and the production of antibiotic-inactivating enzymes and nonenzymatic mechanisms (55). The higher level of MDR might be due to the fact that *Acinetobacter* can produce biofilm. Because bacterial colonies are encased or shielded by an extracellular polymeric substance matrix, they are metabolically limited and resistant to antimicrobials. This pathogen is considered a critical priority on the list of pathogens at the top of the list of novel antibiotics (56, 57). Furthermore, *E. coli*, *Klebsiella* spp. and *Acinetobacter* spp. were the most predominant concurrently isolated multi-drug resistant pathogen from mothers and neonates. These findings suggest the possible cross-transmission of the multidrug-resistant microorganisms (MDROs) to newborns.

Recognizing the risk factors for pathogen colonization in pregnant women and vertical transmission to newborns is crucial to lowering the morbidity and mortality associated with this pathogen’s neonatal diseases. The likelihood of vaginal colonization with a neonatal disease pathogen was increased by the presence of domestic animals in the house (AOR: 2.88, 95% CI: 1.55–5.36, *p* = 0.001). Previous evidence had supported the idea that living with animals would be considered an important risk factor for infection with *E. coli* (58). Additionally, this finding was supported by evidence that living with cats and dogs increases the vaginal colonization with *E. coli* in pregnant women (59). The predominance of *E. coli* in this study might also be linked with such associations. Furthermore, vaginal colonization was significantly associated with urinary tract infections (AOR: 2.66, 95% CI: 1.23–5.76, *p* = 0.013). Like our findings in Jimma, Ethiopia (60), Arbaminch, Ethiopia (51), and Iran (35), GBS maternal colonization was associated with urinary tract infection. This is because the predominant bacteria isolated in this study cause UTIs in pregnancy (61). Those bacteria may spread from the urethra to the vagina. Likewise, the odds of vaginal colonization were significantly higher among women with PROM (AOR: 2.95, 95% CI: 1.29, 6.74, *p* = 0.010). It was supported by two studies from India (62, 63), where GBS maternal colonization was significantly associated with PROM. It is hypothesized that colonization of the vagina with pathogenic bacteria activates the local and upper (cervical and fetal membrane) innate immune systems, driving an inflammatory cascade that leads to remodeling and disruption of membrane architecture and premature rupture (64).

Vertical transmission was independently associated with duration of labor (AOR = 2.23, 95% CI: 1.11, 4.47, *p* = 0.024). The odds of vertical transmission were higher among women whose labor was prolonged for more than 12 h than their counterparts. This was consistent with a study conducted in Tanzania (65), where prolonged duration of labor (>12 h) was significantly shown to influence GBS colonization in neonates. This may be due to prolonged exposure of the neonate to the birth canal. The other factor significantly associated with vertical transmission of potential neonatal pathogens to newborns was the low birth weight of newborns (AOR = 5.38, 95% CI: 2.17, 13.36, *p* ≤ 0.001). Newborns with a low birth weight (<2.5 kg) were more colonized than their counterparts, which was supported by another Ethiopian study done in Arbaminch, Ethiopia (51). Moreover, low birth weight is one of the significant factors in neonatal sepsis (66).

The finding of vaginal colonization and vertical transmission among pregnant women and neonates in this study highlights crucial clinical and public health implications. The identified high level of pathogens and MDR highlight the need for tailored treatment strategies and effective antimicrobial stewardship programs (ASP), identification of pathogens and surveillance systems to combat resistance. Therefore, improvement of maternal and neonatal health outcomes and mitigation of vertical transmission requires implementation of targeted public health intervention and regular health promotion activities from the clinical perspectives. Furthermore, from a public health perspective, the findings strongly stress the urgency of addressing neonatal health issues including antimicrobial resistance surveillance, research, resource allocation, policy development and global health initiatives. Overall, this study provides a critical insight into the complex interplay between maternal colonization, vertical transmission of pathogens, antimicrobial resistance, and associated risk factors among pregnant women and neonates in Northeast Ethiopia. As a limitation, neonatal outcomes are not included in our report since neonates born by the study participants were not included due to constraints. Moreover, we used vaginal sampling instead of recto-vaginal sampling for the detection of GBS (as recommended by the CDC), which has been shown to have higher recovery rates for GBS. We perform AST only for GBS*, S. aureus, E. coli, Acinetobacter* spp., and *Klebsiella* spp. because of antibiotic disc shortage.

## 5 Conclusion

This study revealed a high colonization rate of potential neonatal pathogens in pregnant women and their newborns. *E. coli*, *Candida* spp., and *Klebsiella* spp. were the frequently isolated potential pathogens from both pregnant women and their newborns. This study revealed high level of MDR and antibiotic resistance among potential pathogens, indicating a significant public health challenge. Having domestic animals in the house, history of UTI, and premature rupture of the membrane were significantly associated with pregnant women’s colonization with potential neonatal pathogens. Likewise, duration of labor and weight of newborns were significantly associated with the colonization of potential neonatal pathogens in newborns. The findings of vaginal colonization and vertical transmission were alarming for further longitudinal studies will be required to evaluate the virulence of the potential neonatal pathogens and the risk of neonatal infections as well as to design an appropriate preventive strategy. Furthermore, implementation of effective antimicrobial stewardship programs, and a regular surveillance system are quite advisable to monitor antimicrobial resistance patterns, optimize antibiotic use to curb colonization and transmission, raise awareness about the risks of neonatal infections, and develop policies to address prevention and management of neonatal infections.

## Data Availability

The original contributions presented in the study are included in the article/supplementary material, further inquiries can be directed to the corresponding author.
